# Social isolation postweaning alters reward-related dopamine dynamics in a region-specific manner in adolescent male rats

**DOI:** 10.1016/j.ynstr.2024.100620

**Published:** 2024-03-02

**Authors:** Valeria Lallai, Cristina Congiu, Giulia Craig, Letizia Manca, Yen-Chu Chen, Angeline J. Dukes, Christie D. Fowler, Laura Dazzi

**Affiliations:** a Department of Neurobiology and Behavior, University of California Irvine, Irvine, CA, 92697, USA; b Department of Life and Environmental Sciences, Section of Neuroscience and Anthropology, Centre of Excellence for the Neurobiology of Dependence, University of Cagliari, 09042, Monserrato, CA, Italy

## Abstract

Early development is characterized by dynamic transitions in brain maturation, which may be impacted by environmental factors. Here, we sought to determine the effects of social isolation from postweaning and during adolescence on reward behavior and dopaminergic signaling in male rats. Subjects were socially isolated or group housed at postnatal day 21. Three weeks later, extracellular dopamine concentrations were examined in the medial prefrontal cortex (mPFC) and nucleus accumbens shell (NAc) during a feeding bout. Surprisingly, opposing effects were found in which increased mPFC dopamine concentrations were observed in group housed, but not isolated, rats. In stark contrast, increased dopamine levels were found in the NAc of isolated, but not group housed, rats. Moreover, the absence of an effect in the mPFC of the isolated rats could not be reversed by subsequent group housing, demonstrating the remarkable long-term effects on dopamine signaling dynamics. When provided a highly palatable food, the isolated subjects exhibited a dramatic increase in mPFC dopamine levels when the chocolate was novel, but no effects following chronic chocolate consumption. In contrast, the group housed subjects showed significantly increased dopamine levels only with chronic chocolate consumption. The dopamine changes were correlated with differences in behavioral measures. Importantly, the deficit in reward-related behavior during isolation could be reversed by microinjection of either dopamine or cocaine into the mPFC. Together, these data provide evidence that social isolation from postweaning and during adolescence alters reward-induced dopamine levels in a brain region-specific manner, which has important functional implications for reward-related behavior.

## 1 Introduction

While chronic social isolation and loneliness have been well established to have a negative impact on physical and mental health, the broad impact of school closures and home confinement during the COVID pandemic brought to the forefront the need to better understand such impacts on the developing brain (Agostino et al., 2021; Cortes-Garcia et al., 2022; Hawkley and Cacioppo, 2003; Loades et al., 2020). Social isolation has been correlated with lower life satisfaction, higher levels of substance abuse, depression, anxiety, and increased stress in humans (Clair et al., 2021; Loades et al., 2020). Rodent studies have also shown that social interaction can act as a primary motivating reinforcer (Evans et al., 1994; Novick et al., 2018), whereas social isolation stress can lead to increased depression- and anxiety-associated behaviors, aggression, increased drug self-administration, and impaired learning and memory function (Bianchi et al., 2006; Brenes et al., 2008; Del Arco et al., 2004; Heidbreder et al., 2000; Lallai et al., 2016; McCool and Chappell, 2009; Schenk et al., 1987; Wongwitdecha and Marsden, 1996; Yajie et al., 2005). Such behavioral effects have been correlated with neurobiological changes in the developing central nervous system. For instance, social isolation early in development can lead to long-term alterations at the neurochemical and structural levels (Day-Wilson et al., 2006; Fabricius et al., 2010; Fone and Porkess, 2008; Fone et al., 1996; Heidbreder et al., 2000; Pascual et al., 2007; Scaccianoce et al., 2006; Serra et al., 2000; Silva-Gomez et al., 2003), such as with altered synaptic plasticity and reduced BDNF levels in the hippocampus (Scaccianoce et al., 2006) or reduced dendritic spine density in the medial prefrontal cortex (mPFC) (Day-Wilson et al., 2006; Pascual et al., 2007; Silva-Gomez et al., 2003).

The mPFC receives projections from several brain regions, including dopaminergic axons of the ventral tegmental area (VTA). The mPFC has been shown to play a key role in decision-making and control of behavior, and dysfunction of this area has been linked to impulsivity and disrupted cognitive control, which can underlie the progression of compulsive drug use (Bechara et al., 1999; Murray and Rudebeck, 2018; Peters and Buchel, 2011; Rogers et al., 1999). Interestingly, stress-related events have been shown to activate the VTA-mPFC projecting neurons (Abercrombie et al., 1989; Dazzi et al., 1995; Finlay and Zigmond, 1997; Hamamura and Fibiger, 1993; Inglis and Moghaddam, 1999), and this signaling pathway has been proposed to underlie the pathology associated with mood disorders (McCormick and Green, 2013; Paus et al., 2008). Dopaminergic fibers from the VTA also innervate the nucleus accumbens (NAc). Together, the mPFC and NAc regulate neural processing for the attribution of incentive salience and motivation underlying goal-directed behaviors, along with coordinating the responsiveness to stimuli associated with both food and drugs (Bassareo et al., 2011; Oterdoom et al., 2020; Robinson and Berridge, 1993; Salamone et al., 2007). These two regions are also interconnected, with the mPFC sending excitatory inputs into the NAc to elicit physiological and behavioral responses (Britt et al., 2012). This mPFC-NAc circuit becomes activated during social interactions, and inactivation impairs social recognition (Park et al., 2021). Of relevance, social isolation has been associated with increased dopamine transporter levels, decreased dopamine D2 receptor expression, altered dendritic spine density, and decreased presynaptic excitatory transmission in the NAc (Deutschmann et al., 2022; Lampert et al., 2017; McGrath and Briand, 2022), which demonstrates the impact that social isolation can have on dopaminergic function during periods of neural development. However, the extent of the effects of social isolation on neurotransmitter signaling during adolescence remain largely unknown.

Natural reward consumption represents a basic biological drive to take in nutritive substances to support biological function and health. Food intake has been shown to involve the VTA, mPFC, NAc, amygdala and hippocampus (Bassareo et al., 2011; Higgs, 2016; Meye and Adan, 2014). Intake of palatable foods involves activation of the dopaminergic circuit, which is thought to lead to an increased reinforcing drive that can exceed the basic nutritional needs of the individual (Erlanson-Albertsson, 2005; Hajnal et al., 2004; Lenoir et al., 2007). Indeed, the drive to obtain palatable food can be maintained even under aversive conditions. For instance, rats will continue to exhibit a high motivation to obtain palatable food rewards in the presence of an aversive foot shock (Leigh and Morris, 2018), and mice will spend more time in an adverse environment that was previously associated with palatable food (Teegarden and Bale, 2007). Excessive or insufficient food intake can also become pathological and thus diagnosed as an eating disorder (El Archi et al., 2020; Oterdoom et al., 2018), which can often be comorbid with symptoms of anhedonia (Bolton et al., 2018; Dolan et al., 2022; Murray et al., 2022).

This series of studies sought to examine the impact of social isolation postweaning and during adolescence on reward-related behaviors and dopamine dynamics in the mPFC and NAc shell in male rats. Immediately after weaning, subjects were either socially isolated or group housed. Dopamine concentrations in the mPFC and NAc were examined before, during, and after the presentation of palatable food. Given proposed sex-specific differences in developmental patterning (Andersen et al., 1997; Kopec et al., 2018; McGrath and Briand, 2022) along with sex-specific effects of stress (McGrath and Briand, 2022) within the mesolimbic and mesocortical pathways, we focused herein on males, and thus, future studies will be needed in females. Next, to understand if the isolation-induced changes were reversible, we examined whether group housing would reinstate the altered dopamine levels following 3 weeks of social isolation. Thereafter, to determine whether these changes in dopamine signaling were directly related to reward-seeking behaviors, subjects were then examined for reinforcement behavior with food self-administration. Given that differences were observed supporting the correlation between dopamine levels in the mPFC and reward-related deficits, we then assessed whether these deficits could be ameliorated by site-specific pharmacological modulation of dopamine levels. Taken together, our findings provide evidence that social isolation from postweaning and during adolescence results in opposing effects on the mesolimbic and mesocortical pathways in male rats, and the subsequent deficits in the drive to obtain food reward can be mediated by increasing dopamine levels in the mPFC, an effect that was also induced by cocaine.

## 2 Materials and methods

### 2.1 Animals

Sprague Dawley rats were ordered from Charles River and bred in the laboratory facility. Rats were maintained under a 12 h light, 12 h dark cycle, at a constant temperature of ∼22 °C, and a relative humidity of ∼65%. In total, n = 83 male rats were examined in these studies. All subjects were provided *ad libitum* access to water and standard laboratory chow until the start of behavioral testing, at which time food was restricted, and *ad libitum* access to water was maintained. For the microdialysis studies, the experimental protocols were approved by the Animal Ethics Committee of the University of Cagliari and by the Italian Ministry of Health (authorization #353/2015-PR), and animal care and handling throughout the experimental procedures was conducted in strict accordance with the European Communities Council Directive of November 24, 1986 (86/609/EEC). For the food self-administration and cannulation studies, experiments were approved by the Institutional Animal Care and Use Committee at the University of California Irvine and were conducted in strict accordance with the NIH Guide for the Care and Use of Laboratory Animals.

### 2.2 Experimental design

**Fig. 1:**
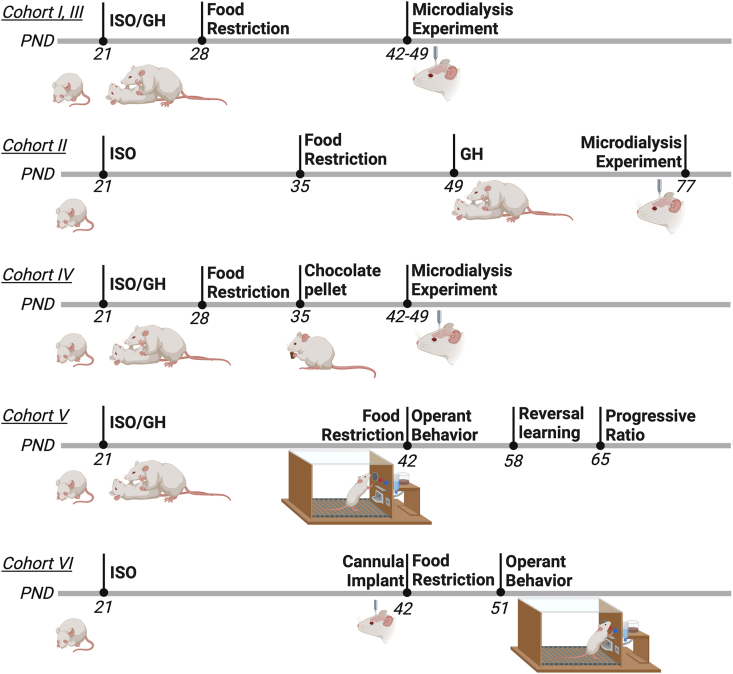
**Timeline of experimental procedures for each cohort of subjects**. Rats were weaned at postnatal day (PND) 21 and then placed into isolated or group housed conditions. Each PND noted indicates when the analyses for each part of the experiment were initiated for each cohort. ISO: Isolated, GH: Group housed. Figure generated with Biorender.com.

Subjects were weaned at postnatal day (PND) 21, and housed individually (socially isolated, ISO) or in groups of 3–5 per cage (group housed, GH). Experimental conditions are outlined in Fig. 1. For Cohorts I-IV, starting at PND28, both isolated and group housed rats began limited feeding access with standard food chow (Standard GLP diet, Mucedola, Italy) available during a restricted 2 h period in their home cages, and this feeding paradigm was maintained throughout the remainder of the experiment. Group housed subjects consumed their daily food with their cage mates, except during the microdialysis experiment in which subjects were single housed to ensure maintained integrity of the dialysis line for sample collection. With the 2 h duration of feeding, the rats continued to increase their weights consistent with that expected based on the vendor growth chart for Sprague Dawley rats, regardless of group condition. To examine the effects of food reward on dopamine signaling between housing conditions, Cohort I and II were implanted with a microdialysis probe into the mPFC, and Cohort III into the NAc. To evaluate if group housing could reverse the effects of social isolation on dopamine levels, Cohort II was isolated for 4 weeks, which corresponds to the full duration of isolation for Cohorts I/III, and then they were returned to group housing conditions for an additional 4 weeks (ISO + GH). Next, to examine whether the availability of a highly palatable food would have a differential effect on dopamine levels, Cohort IV rats were treated similarly to Cohort I/III, but chocolate pellets were made available beginning on PND35 for two weeks before the experiment (chronic, long-term exposure), or only on the day of the experiment (acute exposure). Isolated subjects received the chocolate pellets in their home cages, while group housed subjects were placed in individual cages to ensure full consumption for each animal across the 2 h period; both groups consumed the chocolate provided daily. We next sought to examine whether the differences in dopamine dynamics induced deficits in the motivation to obtain natural reward in Cohorts V and VI. On PND42, subjects were food restricted to allow for training with food self-administration. Cohort V was examined with operant food training beginning at PND42, reversal learning at PND58, and progressive ratio testing at PND65. Finally, to examine whether dopamine specifically in the mPFC regulated the behavioral effects evidenced in the isolated condition, Cohort VI rats were isolated on PND21, implanted with bilateral cannula directed at the mPFC ∼ PND42, and underwent operant food training after 3 days of surgical recovery. After achieving baseline responding, they were then microinjected with cocaine or dopamine prior to daily food self-administration sessions.

### 2.3 Surgical and experimental procedures

#### 2.3.1 In vivo microdialysis

For Cohorts I-IV, rats were anesthetized by intraperitoneal injection of Equithesin (5 ml/kg), and a concentric dialysis probe was inserted at the level of the mPFC (AP +3.2 mm; ML +0.8 mm; DV -5.3 relative to bregma) or NAc shell (AP +2.2 mm; ML +1.0 mm; DV –7.8 mm relative to bregma) (Lallai et al., 2016; Paxinos and Watson, 2005). To allow for recovery from surgical procedures, experiments were performed 24–36 h after probe implantation. Both group and single housed rats were placed alone in microdialysis collection chambers that allowed them to freely move during food consumption and sample collection. Ringer's solution was pumped through the dialysis probe at a constant rate of 2 μl/min. Samples of dialysate were collected every 20 min and immediately analyzed for dopamine with High-Performance Liquid Chromatography (HPLC) with electrochemical detection, as previously described (Dazzi et al., 2002). The detection limit for dopamine was 2 fmol. The average neurotransmitter concentration in the first two samples was taken as 100%, and all subsequent values were expressed relative to the baseline value for each subject. The mean *in vitro* recovery of the probes was 15 ± 3%. All probes were tested before implantation, and those with a recovery value outside of this range were not used. At the end of each experiment, the placement of the probe was verified histologically. Six rats were found to have the probe located outside of the target region or the implant was damaged across time, and thus, they were excluded from the analysis.

#### 2.3.2 Reward-related behavioral responding

For Cohort V, subjects began operant training for food reward at PND42. The pellets provided in the operant chambers were either a standard chow pellet (TestDiet, 5TUM, chow pellet with 10.4% fat content) or a higher fat sucrose chocolate-flavored pellet (TestDiet, 5TUL, sucrose pellet with 12.7% fat content). Following food restriction to 85–90% of their free feeding weight, subjects were trained to press a lever in an operant chamber (Med Associates) with the palatable chow food pellets up to a fixed-ratio 5, time out 20 s (FR5TO20s) schedule of reinforcement. Each session was performed using two retractable levers (one active, one inactive). Completion of the response criteria on the active lever resulted in the delivery of one food pellet. Responses on the inactive lever were recorded but had no scheduled consequences. For the chocolate pellet studies, once stable responding criteria was achieved (>50 pellets per session across 3 sessions), subjects were tested for novel food with the more palatable chocolate food pellets in the operant box hoppers. After at least 3 consecutive sessions, at PND58 the lever assignment was switched to examine reversal learning, in which the previous inactive lever became active and food pellets were earned in accordance with the established FR5TO20s schedule. In contrast, the previously active lever became inactive, in which responses were recorded but without scheduled consequence. The reversal learning test has been commonly used to assess cognitive flexibility across species (Izquierdo et al., 2017; Sherafat et al., 2021). Behavioral responses were automatically recorded by Med Associates software. Following the reversal test, the animals were provided daily access to re-establish the original active and inactive lever responding at a FR5TO20s schedule of reinforcement for 7 baseline days. At PND65, subjects were tested for the motivational state of acquisition of food in the progressive ratio paradigm. Specifically, to obtain the chocolate pellet rewards, the animals were required to press the active lever with a logarithmic increase in the ratio and the progression of response requirements was as follows: 5, 10, 17, 24, 32, 42, 56, 73, 95, 124, 161, 208, 268, 345, 445, 573, 737, 947, 1218, 1566. Maximum time was set to 6 h for the progressive ratio session.

#### 2.3.3 In vivo pharmacological injections with reward-related responding

For Cohort VI, rats were anesthetized with isoflurane, and 23-gauge stainless steel bilateral cannulas were implanted into the mPFC (AP +3.2 mm; ML ± 0.8 mm; DV -4.3 relative to bregma). Following 72 h of post-surgical recovery, subjects were operantly trained for food self-administration as described above with food restriction to 85–90% of their free feeding weights. Once stable responding was achieved at the FR5 TO20 s schedule of reinforcement, intra-mPFC drug infusions were administered with an injector extended 1 mm below the tip of the cannula (2 min infusion, 1 μl/min rate of infusion) immediately prior to the self-administration session. To examine whether mPFC infusion of dopamine could reinstate food responding in isolated subjects, dopamine hydrochloride (Sigma Aldrich) was administered at a concentration of 500 mM, which is consistent with the concentration observed in the group housed subjects in mPFC dialysate. Given positive findings with dopamine infusions, we next examined whether similar effects could be induced with cocaine, which increases synaptic dopamine levels by inhibiting the actions of the dopamine transporter; cocaine hydrochloride (Sigma Aldrich) was administered at a concentration of 2 mM. Given that dopamine reuptake or degradation can occur relatively rapidly, these food self-administration sessions were restricted to 15 min following the mPFC infusions.

### 2.4 Statistical analysis

Data were analyzed by a *t*-test, one-way or two-way ANOVA with Prism 10 software (GraphPad, La Jolla, CA, USA), as appropriate. Data obtained across sessions were analyzed with a repeated measures ANOVA; significant main or interaction effects were followed by Bonferroni or Tukey post-hoc comparison with correction for multiple comparisons. The criterion for significance was set at α = 0.05.

## 3 Results

### 3.1 Effects of housing condition and standard chow on extracellular dopamine

**Fig. 2:**
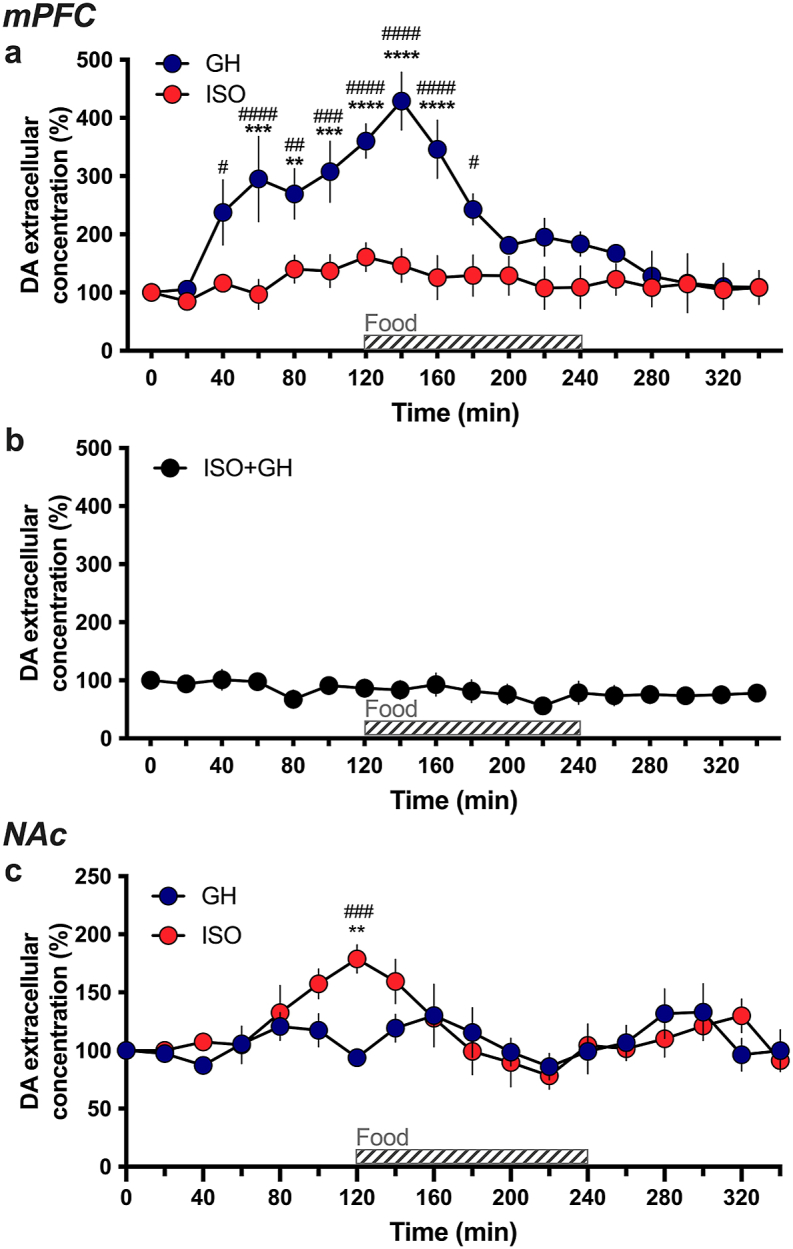
**Opposing effects of feeding on dopamine extracellular concentrations in the mPFC and NAc of group housed or socially isolated rats**. Group housed and isolated subjects received standard food chow for 2 h every day across 3 weeks, and then microdialysis samples were collected on the final day across every 20 min. **(a)** In the mPFC, group housed subjects exhibited a dramatic increase in dopamine concentrations in the anticipatory and food consumption stages, whereas the isolation subjects did not differ from baseline levels (n = 6 per housing condition). **(b)** A group of isolated rats was then group housed for an additional 4 weeks following the isolation period, but this reinstated group housing condition did not lead to food consumption subsequently altering dopamine release in the mPFC (n = 6). **(c)** In the NAc shell, the isolated, but not group housed, subjects exhibited an increase in dopamine concentrations with food consumption (n = 6–7 per housing condition). **p < 0.01, ***p < 0.001, ****p < 0.0001 vs baseline levels; ^#^p < 0.05, ^##^p < 0.01, ^###^p < 0.001, ^####^p < 0.0001 ISO vs GH. ISO: isolated, GH: group housed, ISO + GH: Isolated and then group housed, DA: dopamine. Data are expressed as mean percentage of baseline values ± SEM.

In the mPFC, group housed rats fed under chronic restricted conditions exhibited an increase in extracellular dopamine concentrations as early as 80 min prior to food presentation and levels further increased during food intake to reach a maximal value of ∼420% at 20 min post-food presentation (Fig. 2a). Thereafter, dopamine levels returned to baseline values ∼40 min after the feeding bout. Surprisingly, in socially isolated rats, no substantial changes were observed in mPFC extracellular dopamine concentrations throughout the feeding bout. Comparisons between the group housed and isolated subjects demonstrated significant differences (Repeated measures two-way ANOVA, *Housing* F_(1,110)_ = 11.60, p = 0.0067, *Time* F_(17,170)_ = 8.122, p < 0.0001, *Interaction* F_(17,170)_ = 4.685, p < 0.0001). Specifically, the post-hoc revealed a statistically significant increase from baseline levels for the group housed subjects immediately prior to food (time 60 min, p = 0.0005; 80 min, p = 0.0057; 100 min, p = 0.0001), and during the earlier phase of the feeding session (time 120 min, p < 0.0001; 140 min, p < 0.0001; 160 min, p < 0.0001). Compared to the socially isolated subjects, the group housed subjects also had significantly higher dopamine levels during the anticipatory period (time 40 min, p = 0.0132; 60 min, p < 0.0001; 80 min, p = 0.0085; 100 min, p = 0.0006) and during the earlier phase of feeding (time 120 min, p < 0.0001; 140 min, p < 0.0001; 160 min, p < 0.0001; 180 min, p = 0.0204). We next evaluated whether the blunted response of the mesocortical dopaminergic neurons in isolated subjects could be reversed by a socially enriched environment. Therefore, after the same isolation duration, a group housing condition was reinstated for 4 consecutive weeks, with subjects returned to cages containing their prior cage-mates (Fig. 2b). Surprisingly, exposure of the previously isolated animals to a socially enriched environment failed to restore the sensitivity of mesocortical dopaminergic neurons to food reward (One-way ANOVA, F_(17,90)_ = 0.6138, p = 0.8730). Further, when comparing to the isolation group data shown in Fig. 2a, no statistically significant differences in mPFC dopamine concentrations were found between the socially isolated subjects and the subjects reinserted into the group housing condition after isolation (Repeated measures two-way ANOVA, *Housing* F_(1,10)_ = 2.616, p = 0.1368, *Time* F_(17,170)_ = 0.8138, p = 0.6757, *Interaction* F_(17,170)_ = 1.040, p = 0.4183). Thus, these findings indicate that social isolation postweaning and during adolescence disrupted the reward-associated increase in dopamine levels in the mPFC and that subsequent group housing is insufficient to reverse these effects on the reward circuitry. Importantly, no differences were found in body weight between groups (Mean ± SEM, GH 259.4g ± 10.55 vs. ISO 259.7g ± 6.016; *t*-test, t_(42)_ = 0.0242, p = 0.9808), which provides evidence that group differences were not due to varying hunger drives, but rather, were reward-related.

Next, we sought to investigate whether changes in dopamine levels could be detected in other regions of the brain reward circuit. We hypothesized that plasticity in circuit function due to social isolation could have occurred, leading to changes in dopamine release in other dopaminergic-signaling regions. To this end, extracellular dopamine concentrations were assessed in the shell of the NAc in rats under the conditions as above in Fig. 2a. Interestingly, we found that in socially isolated rats, extracellular dopamine concentrations significantly increased as early as 20 min before food to reach a maximal value of ∼180%, and then returned to baseline values 20 min after food removal (Fig. 2c). In striking contrast, group housed rats exhibited no significant changes in dopamine concentrations in the shell of the NAc across time (Repeated measures two-way ANOVA, *Housing* F_(1,11)_ = 0.4406, p = 0.5205, *Time* F_(17,187)_ = 3.737, p < 0.0001, *Interaction* F_(17,187)_ = 2.218, p = 0.0050). The post-hoc analyses revealed that the isolated group exhibited significantly higher levels of NAc dopamine levels as compared to their own baseline levels at time 120 (p = 0.0040), and as compared to the group housed subjects at time 120 (p = 0.0001). No differences were found in body weight between groups (GH 239.8g ± 10.55 vs. ISO 235.6g ± 6.016; *t*-test, t_(42)_ = 1.010, p = 0.3182). These findings reveal a dissociation in reward-related dopamine dynamics in a brain region specific manner due to social environment conditions from postweaning age.

### 3.2 Effect of palatable food on mPFC extracellular dopamine concentrations

**Fig. 3:**
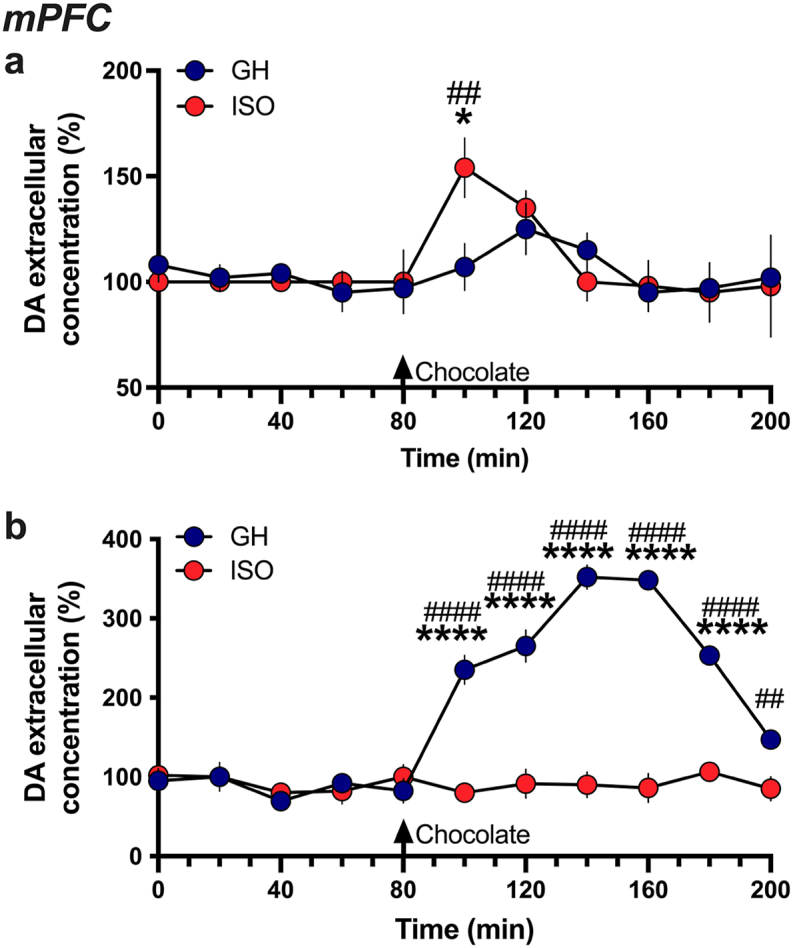
**Consumption of highly palatable chocolate differentially increases mPFC dopamine levels based on housing conditions and food novelty**. **(a)** Dopamine levels were examined during the presentation of novel chocolate food pellets. The isolated, but not group housed, subjects exhibited a significant increase in mPFC dopamine concentrations (n = 6 per housing condition). **(b)** After having access to consume chocolate pellets across 2 weeks, the group housed, but not isolated, subjects exhibited a dramatic increase in dopamine concentrations (n = 6 per housing condition). *p < 0.05, ****p < 0.0001 vs baseline; ^##^p < 0.01, ^####^p < 0.0001 ISO vs GH. ISO: isolated, GH: group housed, DA: dopamine. Data are expressed as the mean percentage of baseline values ± SEM.

To examine whether a food with higher incentive salience could induce differential effects on dopamine release related to social environment, we next monitored extracellular dopamine concentrations in the mPFC during acute chocolate pellet exposure and consumption. Interestingly, socially isolated rats exhibited a significant increase in their extracellular mPFC dopamine concentrations during the chocolate consumption, which was greater than that found in group housed subjects (Repeated measures two-way ANOVA, *Housing* F_(1,110)_ = 0.4581, p = 0.4999, *Time* F_(10,110)_ = 2.920, p = 0.0028, *Interaction* F_(10,110)_ = 1.195, p = 0.3022; post-hoc: Isolation vs. baseline 100 min, p = 0.0156; GH vs ISO 100 min, p = 0.0018) (Fig. 3a). Given that this was the first exposure to chocolate pellets, the effects evidenced could arguably be due to novelty or a higher rewarding value of palatable food. Thus, subjects were then provided access to the chocolate pellets every day for two weeks. Surprisingly, following this long-term access to chocolate pellets, group housed rats exhibited a dramatic increase in extracellular dopamine concentrations in the mPFC, an effect that was not observed in the socially isolated subjects (Two-way ANOVA, *Housing* F_(1,110)_ = 243.1, p < 0.0001, *Time* F_(10,110)_ = 29.06, p < 0.0001, *Interaction* F_(10,110)_ = 29.84, p < 0.0001) (Fig. 3b). The post-hoc analyses revealed significant increases in dopamine levels from baseline for the group housed subjects during food consumption at times 100 min (p < 0.0001), 120 min (p < 0.0001), 140 min (p < 0.0001), 160 min (p < 0.0001), and 180 min (p < 0.0001). Significant differences between the groups were also found with the group housed subjects having higher mPFC dopamine levels than isolated subjects at times 100 min (p < 0.0001), 120 min (p < 0.0001), 140 min (p < 0.0001), 160 min (p < 0.0001), 180 min (p < 0.0001), and 200 min (p = 0.0025). Importantly, when we compared the weights of the subjects, no differences were found between groups (GH 255.0g ± 7.96 vs ISO 244.7g ± 5.35; *t*-test, t_(42)_ = 1.072, p = 0.2899), and all the subjects consumed the full amount of chocolate pellets provided, indicating that subject weight or total food consumed was not a factor in this difference. Together, these findings demonstrate that socially isolated animals exhibit a deficit in signaling for familiar palatable food, as evidenced by a lack of increased dopamine release in the mesocortical pathway with chronic chocolate consumption.

### 3.3 Effect of social environment on reward behavior

**Fig. 4:**
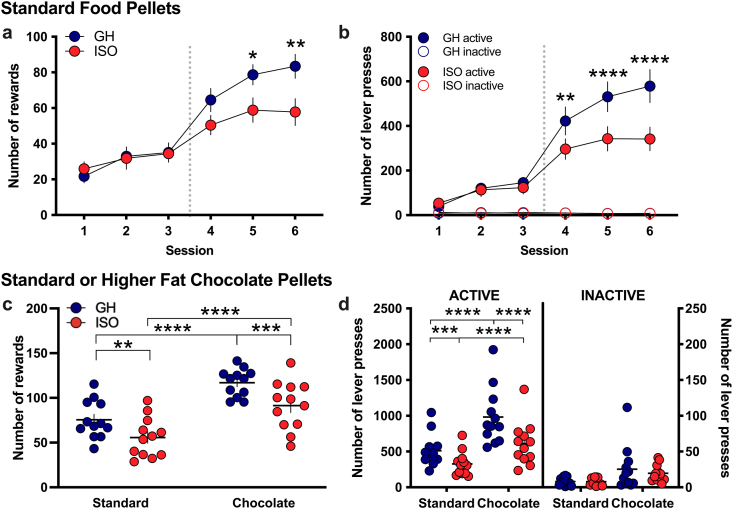
**Social isolation attenuates the reinforcing value of natural reward with both chow and higher fat, palatable chocolate pellets**. **(a)** Subjects were examined for their ability to learn an operant task to obtain standard chow food rewards under an ascending fixed ratio schedule of reinforcement (n = 12 per housing condition). All subjects achieved the final FR5 reinforcement schedule on session 3, which was maintained across sessions 4–6. While both groups exhibited similar initial learning up to the FR5, the isolated subjects diverged to earn fewer self-administered food rewards while maintaining the FR5 schedule on sessions 4–6. **(b)** Examination of the number of active and inactive lever presses reveals a significant difference in active lever presses between the isolated and group housed subjects across sessions 4–6, whereas no differences were observed between groups or across sessions for the inactive lever pressing behavior. **(c)** When more palatable chocolate pellets were substituted as reward, both groups increased the number of self-administered rewards. However, the isolated subjects continued to earn significantly less rewards than the group housed subjects, for both the chow and chocolate pellets. **(d)** Similar differences were found for the active lever as that observed with the number of food pellet rewards earned. No differences were found between groups or food conditions for the number of inactive lever presses. *p < 0.05, **p < 0.01, ***p < 0.001, ****p < 0.0001 ISO vs GH. ISO: isolated, GH: group housed. Data represent mean ± SEM.

Given the differences in dopamine dynamics in the mPFC and NAc due to social environment, we next examined whether the groups would differ in their motivation to obtain food reward with an operant self-administration procedure. Subjects were first trained to self-administer standard food chow pellets in operant boxes from a fixed ratio 1 up to a fixed ratio 5, time out 20 s (FR5 TO20 s) schedule of reinforcement. Subjects in both groups similarly learned the food training task across the first three days of training, in which all subjects achieved the criteria of >35 rewards at the FR5TO20 s reinforcement schedule. As the groups stabilized, significant differences emerged in the number of food rewards earned (Repeated measures two-way ANOVA, *Housing* F_(1,11)_ = 2.994, p = 0.1115, *Time* F_(5,55)_ = 59.91, p < 0.0001, *Interaction* F_(5,55)_ = 3.290, p = 0.0114) (Fig. 4a). The post-hoc test revealed significant dissociation based on social environmental housing condition for session 5 (p = 0.0252) and session 6 (p = 0.0018). This difference was also reflected in the number of presses on the active lever (Repeated measures two-way ANOVA, *Housing* F_(3,33)_ = 36.75, p < 0.0001, *Time* F_(5,55)_ = 101.6, p < 0.0001, *Interaction* F_(15,165)_ = 21.99, p < 0.0001), in which the post-hoc indicated a significant dissociation between groups for session 4 (p = 0.0017), session 5 (p < 0.0001), and session 6 (p < 0.0001) (Fig. 4b). The groups did not differ in their responses on the inactive lever, and both groups exhibited statistically significant preference for the active lever compared to the inactive lever for sessions 2 (p < 0.05), 3 (p < 0.001), and 4–6 (p < 0.0001). Importantly, no differences were found in body weights between groups (GH 240.0g ± 8.18 vs. ISO 232.9g ± 8.65; *t*-test, t_(54)_ = 0.5937, p = 0.5552). These results demonstrate that socially isolated animals had an overall lower drive to obtain food reward than the group housed subjects, findings that are consistent with the lack of a change in mPFC dopamine levels during chow food presentation in the socially isolated group.

To further determine whether these findings extend to palatable food, subjects were permitted to lever press for higher-fat, chocolate pellets. Differences in the number of rewards obtained across sessions and between groups were observed (Repeated measures two-way ANOVA, *Housing* F_(1,11)_ = 6.448, p = 0.0275, *Food type* F_(1,11)_ = 193.8, p < 0.0001, *Interaction* F_(1,11)_ = 1.080, p = 0.3211) (Fig. 4c). Specifically, the post-hoc revealed that the socially isolated subjects earned significantly less pellets than the group housed subjects when provided either standard chow pellets (p = 0.0019) or chocolate food pellets (p = 0.0002). When comparing within groups, the group housed subjects earned more food pellets when chocolate was available (p < 0.0001), similar to that found within the isolated subjects (p < 0.0001). In accordance with the number of rewards earned, similar differences were found for the number of active and inactive lever presses (Repeated measures two-way ANOVA, Housing F_(3,33)_ = 45.34, p < 0.0001, Food type F_(1,11)_ = 97.26, p < 0.0001, Interaction F_(3,33)_ = 39.39, p < 0.0001) (Fig. 4d). The post-hoc revealed that the socially isolated subjects pressed the active lever less than the group housed subjects when provided either standard chow pellets (p = 0.0003) or chocolate food pellets (p < 0.0001). When comparing within groups, more active lever presses were performed when chocolate was available for both the group housed (p < 0.0001) and isolated subjects (p < 0.0001). Within groups and food type, all subjects had significantly more lever presses on the active lever than inactive lever for both conditions (p < 0.0001), and no group differences were observed for the inactive lever alone.

### 3.4 Effect of social isolation on reversal learning

**Fig. 5:**
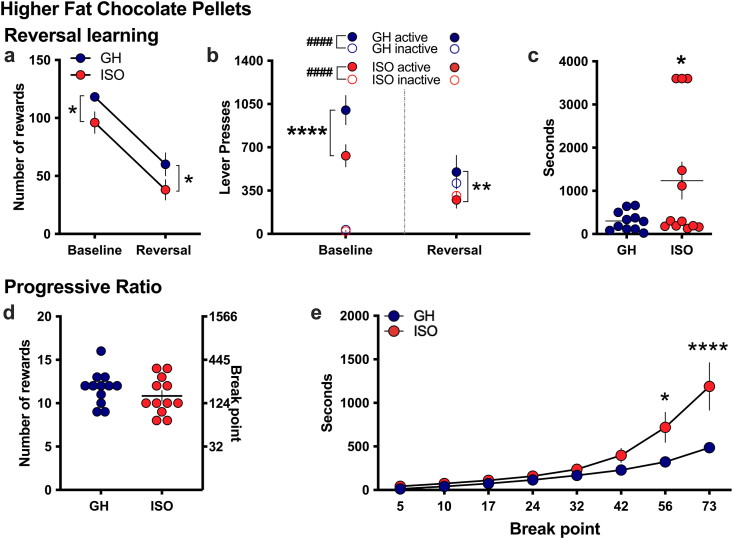
**Social isolation leads to differences in reversal learning and motivation to obtain reward**. **(a)** As a measure of cognitive flexibility, rats were required to reverse their lever pressing behavior with the active and inactive lever assignment switched (n = 12 per housing condition). While both groups reduced the number of rewards earned, the decreased reinforcement behavior was maintained for the isolated subjects, which was lower than the group housed subjects. **(b)** For lever presses, active lever presses were lower in the isolated subjects, compared to the group housed. On the reversal session, the isolated and group housed subjects maintained this difference with the lever assignment switched. **(c)** When examining the latency to receive the first reward on the reversal session, the isolated group exhibited an overall increased latency time to first reward. **(d)** For the progressive ratio test, isolated and group housed subjects did not differ in the total number of rewards earned or break point ratio. **(e)** In examining the latency to achieve each break point ratio, the isolated subjects exhibited an increased latency to achieve the more effortful ratios. *p < 0.05, **p < 0.01, ****p < 0.0001 ISO vs GH. ISO: isolated, GH: group housed. Data represent mean ± SEM.

After establishing consistent responding on the active lever, reversal learning was examined with the higher fat chocolate food pellets at PND58. In this cognitive task, the subjects are required to switch their lever pressing behavior, as the active and inactive lever assignments become reversed in the operant chamber. We observed an overall reduction in the number of rewards obtained for the reversal session compared to the baseline for both experimental groups. This downward trend is reflected in the number of food pellet rewards earned (Repeated measures two-way ANOVA, *Housing* F_(1,11)_ = 4.595, p = 0.0553, *Session* F_(1,11)_ = 53.89, p < 0.0001, *Interaction* F_(1,11)_ = 0.0005, p = 0.9820; post-hoc: Baseline GH vs ISO, p = 0.0292; Reversal GH vs ISO, p = 0.0312) (Fig. 5a). When analyzing lever pressing behavior, the within-session lever pressing differed between the groups on the baseline and reversal session, and no differences were found in inactive lever pressing (Repeated measures two-way ANOVA, *Housing* F_(3,33)_ = 15.15, p < 0.0001, *Session Lever* F_(1,11)_ = 3.782, p = 0.0778, *Interaction* F_(3,33)_ = 48.00, p < 0.0001); Post-hoc, *Baseline*: GH active vs ISO active, p < 0.0001, GH active vs GH inactive, p < 0.0001, ISO active vs ISO inactive, p < 0.0001; *Reversal*: GH active vs ISO active, p = 0.0066) (Fig. 5b). To provide further insight into the animal's flexibility with the shift in procedural requirements, we next analyzed the latency time to obtain the first reward on the newly active lever. The isolated group exhibited a significantly longer latency to achieve the first reward as compared to the group housed rats (*t*-test, t_(21)_ = 2.061, p = 0.0260, R^2^ = 0.1682) (Fig. 5c). This demonstrates that while the subjects were able to adjust their behavioral responding across the 1 h session, the isolated subjects tended to exhibit a delay in initially orienting to the task requirements during the challenge, which may be reflective of an attentional delay.

To further investigate the motivation of the groups to obtain the palatable food reward, subjects were then examined with the progressive ratio schedule of reinforcement, in which each subsequent reward requires increasing demand for lever pressing behavior. This test was performed at PND65, after re-established baseline responding with the original FR5TO20s schedule of reinforcement. Differences between the group housed and isolated subjects were not found in the overall number of rewards obtained (*t-test*, t_(22)_ = 1.111, p = 0.2787, R^2^ = 0.0531) (Fig. 5d), demonstrating that the isolated subjects can respond at the level of the group housed and do not exhibit general movement deficits. We then examined the data for the time each group took to achieve each additional breakpoint ratio across the session. Interestingly, a difference between groups emerged as subjects were required to press for the higher breakpoint ratios (Repeated measures two-way ANOVA, *Housing* F_(1,22)_ = 6.471, p = 0.0185, *Time* F_(7,154)_ = 29.12, p < 0.0001, *Interaction* F_(7,154)_ = 5.573, p < 0.0001) (Fig. 5e). The post-hoc test revealed that the group housed subjects achieved the 56 (p = 0.0102) and 73 (p < 0.0001) breakpoints significantly quicker than the isolated subjects. This analysis suggests that the isolated subjects may have exhibited an underlying disruption in sustained attention across the session duration (Krupina et al., 2020), a finding consistent with the deficits in dopaminergic signaling observed with isolation.

### 3.5 Increasing mPFC dopamine reverses the isolation-induced reward-seeking deficit

**Fig. 6:**
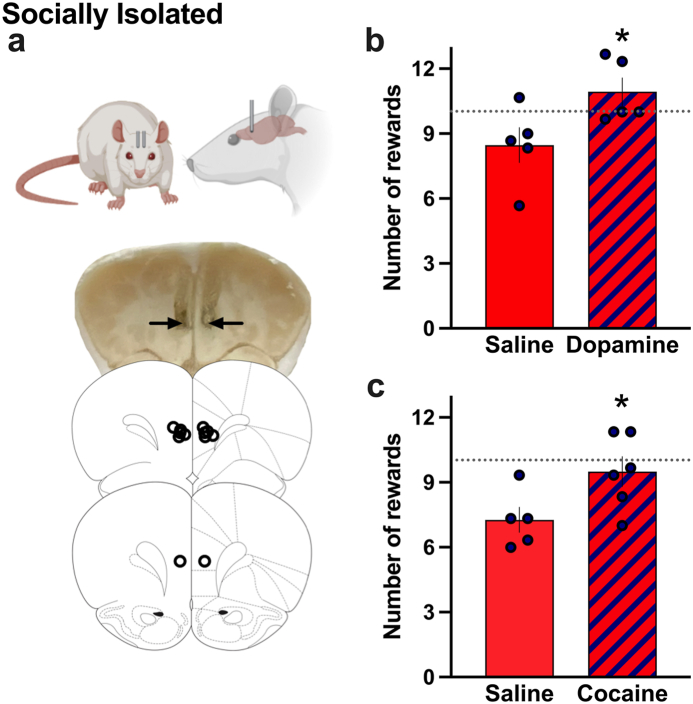
**Normalized reward-seeking behavior in isolated rats achieved by increasing dopamine signaling in the mPFC**. Isolated adolescent subjects were implanted with bilateral cannula directed at the mPFC and then microinjected with dopamine or cocaine to examine reward seeking behavior (n = 5–6 per treatment condition). **(a)** Photomicrograph shows representative image of bilateral cannula placement in the prelimbic area of the mPFC; arrows denote region of infusion. The lower images of brain plates indicate bilateral locations of cannula tips (denoted as black circles), which was verified for each subject in the study. **(b)** Infusion of dopamine into the mPFC significantly increased responding for food reward compared to saline control across the 15 min session. **(c)** Infusion of cocaine into the mPFC also induced an increase in responding for food reward compared to the saline infusion. Of note, the mPFC dopamine- or cocaine-induced level of responding was similar to that found in the previously tested group housed animals in the first 15 min of responding (dotted line). *p < 0.05. Data represent mean ± SEM.

To determine whether the levels of dopamine in the mPFC were directly causing the reward-driven behavioral differences between groups, we next examined whether increasing the level of extracellular dopamine would mitigate the reward-related deficit observed in the isolated rats. Subjects were implanted with bilateral cannula aimed at the mPFC; the locations were validated as accurate following the experiment (Fig. 6a). After achieving a baseline level of responding for the standard chow food pellets, subjects were microinjected immediately prior to the session. Given that dopamine reuptake or degradation can occur relatively rapidly, these food self-administration sessions were restricted to 15 min following the mPFC infusions. In isolated subjects, dopamine administration into the mPFC significantly increased the animal's responding compared to the vehicle control (*t*-test, t_(8)_ = 2.388, p = 0.0440, R^2^ = 0.4162) (Fig. 6b). Next, we examined whether cocaine would induce similar effects when injected directly into the mPFC. Cocaine microinjection into the mPFC led to a statistically significant increase in the number of food pellet rewards compared to the vehicle (*t*-test, t_(9)_ = 2.406, p = 0.0395, R^2^ = 0.3915) (Fig. 6c). When these data are compared to the mean of the group housed animals in the prior study for the first 15 min of the session, the level of reward responding was similar to that found with dopamine or cocaine infusion.

## 4 Discussion

In these studies, we examined the effects of social isolation from postweaning until adolescence on the interplay between dopamine dynamics and reward-driven behavior. Subjects housed in group conditions showed a significant increase in extracellular dopamine levels in the mPFC, but not NAc, during food anticipation and consumption. In contrast, subjects housed in isolation exhibited a significant increase in extracellular dopamine levels in the NAc, but not mPFC, with food consumption. These opposing effects on dopamine release dynamics were further reflected with consumption of a more palatable, high fat diet. Specifically, with acute chocolate, mPFC dopamine levels increased in the isolated, but not group housed, subjects. Conversely, with chronic chocolate consumption, a dramatic increase in dopamine levels was found in the mPFC of the group housed, but not isolated, subjects. The changes in dopamine dynamics between groups was further reflected in motivated behavior to obtain food reward, in which the isolated subjects exhibited a deficit in the overall drive to obtain food reward and motivational delays with effortful cognitive challenge. Importantly, the deficit in reward-related responding was restored with infusion of either dopamine or cocaine into the mPFC, further reinforcing dopamine's actions in this brain structure as underlying the behavioral deficit evidenced in response to social isolation.

### 4.1 Early life as a sensitive period for dopamine system development

In the PFC, dopaminergic innervation from the VTA increases up to threefold during adolescence, reaching a peak around PND60 (Hoops et al., 2018; Naneix et al., 2012; Reynolds and Flores, 2021; Reynolds et al., 2018). In addition, a doubling in the size of axonal dopamine-enriched varicosities occurs from PND20 to PND60, which further corresponds to increases in extracellular dopamine concentrations and dopamine receptor mRNA expression during adolescence (Benes et al., 1996; Naneix et al., 2012; Reynolds and Flores, 2021). This process drives plasticity to promote structural maturation of the PFC pre- and post-synaptic circuit (Reynolds and Flores, 2021; Reynolds et al., 2018). Prior to PND36, D1 receptors are mainly responsible for driving the inhibitory tone of the PFC through activation of GABAergic neurons, whereas D2 receptors have minimal influence on circuit activity (Gorelova et al., 2002; Tseng and O'Donnell, 2007). In contrast, after adolescence, D2 receptor signaling dominates in setting the GABAergic inhibitory tone of the mature PFC, whereas D1 receptors localized on pyramidal neurons modulate excitatory glutamatergic signaling (Naneix et al., 2012; Reynolds and Flores, 2021; Tseng and O'Donnell, 2005, 2007). Importantly, dopaminergic fiber innervation in the NAc and PFC are interrelated, with competing developmental patterning leading to preferential axonal innervation of either the mPFC or NAc (Cuesta et al., 2020; Reynolds et al., 2018). Our findings suggest that social isolation postweaning may disrupt innervation of the mPFC, leading to potentially increased early termination of dopamine terminals in the NAc. Evidence for this may be reflected in the isolated group which exhibited minimal reward-induced dopamine release in the mPFC from baseline levels, which was strikingly contrasted by increased dopamine release in the NAc. Moreover, these changes in mPFC reward-related responsivity with dopamine signaling were persistent, since the effects were unable to be reversed by the subjects' return to a group housed social condition following social isolation.

In the NAc, dopaminergic fiber innervation appears to be mostly established prior to PND20, but dopamine production and corresponding cell spine complexity and dopamine receptor expression continues to increase throughout development into adulthood (Reynolds and Flores, 2021). Indeed, this gradual increase in dopamine signaling during adolescence within the NAc is thought to be critical for the maturation of medium spiny neurons, as spine density becomes established and D2 receptor signaling switches from inhibitory to facilitatory (Lieberman et al., 2018; Reynolds and Flores, 2021; Tepper et al., 1998). This process is paralleled by a peak in striatal D1 and D2 receptors in mid-adolescence, followed by pruning and declining receptor expression until relatively stable levels are observed in adulthood as baseline dopamine levels remain high (Reynolds and Flores, 2021). Therefore, based on the evidence from our studies, social isolation appears to have disproportionately enhanced reward-related dopamine dynamics in the NAc in response to a natural reward, as compared to the group housed condition. Interestingly, this isolation-mediated effect on dopamine signaling likely explains the previously documented increased reinforcing effects of cocaine, amphetamine, and ethanol found with self-administration in isolated rats (Lallai et al., 2016; McCool and Chappell, 2009; Yajie et al., 2005).

### 4.2 Social isolation-induced stress

Early life stressors can disrupt normal neurodevelopmental processes, and this has been demonstrated across species, including rodents and humans (Bolton et al., 2018; Fone and Porkess, 2008; Short and Baram, 2019). While acute stress is thought to be important in allowing an organism to appropriately adapt to varying environmental stimuli, chronic stress can compromise both physiological and behavioral function, especially during developmentally sensitive time periods (Bolton et al., 2018; Fone and Porkess, 2008; Short and Baram, 2019). Indeed, social isolation can be considered a multi-faceted stimulus that induces an impoverished environment through the lack of social interaction and related enriching experiences, thereby increasing the likelihood of an emotional deficit state representative of anhedonia (Olson et al., 2021; Sagud et al., 2021). In rodents, an indicator of an anhedonic state can be a reduction in consumption of palatable food (Birnie et al., 2022; Matthews et al., 1995). The decreased response for food reward observed in the isolated subjects in our study provides evidence that such social isolation is a significant stressor during development, leading to an anhedonic-like state with reduced motivation to obtain food reward. Interestingly, these differences were consistent for standard chow or high fat chocolate pellets, suggesting a pervasive effect regardless of the incentive value of the food. Moreover, examination of the dopamine levels reveals a biological basis for this difference in the reinforcing value of the reward, given that a highly palatable chocolate pellet did not induce a significant change in dopamine release in the mPFC in the isolated subjects. A subtle difference in reversal learning was also found with social isolation, as evidenced by the increased latency time to the first reward in the reversal task. Prior studies have found that an impairment of cognitive flexibility is mainly regulated by the mesocortical dopaminergic neurons that project to the mPFC (Floresco, 2013; Powell et al., 2015). Finally, as noted above, the impact of social isolation on mPFC circuit function may extend to impact other stimuli, including drugs of abuse (Hamamura and Fibiger, 1993; Lallai et al., 2016; Yajie et al., 2005). This hypothesis is further reinforced by the fact that infusion of cocaine into the mPFC normalized the reinforcement behavior of the isolated subjects to group housed levels in our study, thereby supporting the notion that an individual with such altered mPFC signaling could conceivably turn to cocaine use to ‘restore’ circuit activity.

Moreover, given that the isolated subjects showed a significant increase in dopamine levels in the shell of the NAc with acute presentation of the novel palatable food, this pathway appears to predominate in processing the rewarding value of stimuli for this group condition. The shell of the NAc has been shown to regulate processing for outcome, but not predictive, value of rewards, in addition to risk-taking behavior (Freels et al., 2020; Sackett et al., 2017, 2020). Interestingly, increased phasic release of dopamine in the NAc shell is associated with a risk-taking phenotype, as opposed to a risk-averse phenotype (Freels et al., 2020). Together, this suggests that social isolation in the early phase of life may preferentially bias subjects to engage in more risky behavior given the enhanced reward-associated dopamine signaling in this brain region.

It may be further expected that such isolation-mediated alterations of dopamine signaling could lead to additional changes in the postsynaptic neurons. Indeed, early life stress has been found to alter PFC and NAc neuroplasticity during development (McGrath and Briand, 2022; Medendorp et al., 2018; Muhammad et al., 2012; Silva-Gomez et al., 2003; Wang et al., 2012), although the findings thus far have revealed mixed results. For instance, in the PFC, several studies have found reduced dendritic complexity in the PFC with at least 8 weeks of postweaning social isolation in rats and mice (Medendorp et al., 2018; Silva-Gomez et al., 2003; Wang et al., 2012), but findings by Jenrow and colleagues (Medendorp et al., 2018) revealed that the effects were dependent on the type of dendritic spine with an increase in thin spines but a decrease in stubby spines. In contrast, a more recent study that employed AAV expression of GFP to visualize the spines found that postweaning social isolation increased spine density for all of the spine types examined in the PFC of male and female mice, with greater effects in the infralimbic cortex of females (McGrath and Briand, 2022). In this same study, McGrath and Briand (2022) found that postweaning isolation induced an increase in NAc spine density in male, but not female, mice; however, this contrasts with a prior finding in male rats in which isolation was correlated with a reduction in dendritic length, but no effect on spine density (Wang et al., 2012). Given that AAV viral vectors may preferentially infect certain cell types, these data suggest that changes in dendritic complexity may be subregion, cell-type and/or projection specific, which will need to be more systematically investigated in future studies. Taken together, these findings provide evidence that altered dopamine signaling with social isolation at an early developmental stage can alter neuroplasticity.

### 4.3 Persistence of isolation-induced dysregulation

In these studies, we also found that the isolation-induced deficit persisted into adulthood (up to PND77) and was unable to be rescued by re-exposure of the subjects to group housed conditions beginning in late adolescence at PND49. These findings were surprising given that a prior study has shown that rats previously isolated and then put back into groups exhibited an increase in previously reduced neurotrophic factors and increased dendritic spine density in the hippocampus (Biggio et al., 2019). Interestingly, we did observe an increase in mPFC dopamine release in the isolated subjects with acute exposure to a novel palatable food, an effect not found with chronic consumption of the same palatable food. This indicates that dopamine release in the mPFC can be increased in these subjects under some conditions. Furthermore, considering that the avoidance of unfamiliar food is a survival mechanism since unknown food may lead to illness or even death, the decision to consume a novel food may be anxiety- or stress-inducing in rodents (De Oliveira Sergio et al., 2021; Greiner and Petrovich, 2020; Lallai et al., 2022). As such, the increased dopamine release in the isolated subjects only with novel, but not chronic, chocolate availability may be reflective of a higher reactive state in the isolated subjects and/or altered vulnerability to additive stressors (Freels et al., 2020).

### 4.4 Considerations based on sex and other factors

In these studies, we focused on male rats given that the current foundational understanding of early dopamine developmental processes has been derived largely from male subjects (Reynolds and Flores, 2021), and some evidence suggests that different developmental patterning may predominate in females (Andersen et al., 1997; Kopec et al., 2018; McGrath and Briand, 2022). Specifically, it has been shown that females exhibit ∼20% less innervation of dopaminergic fibers into the PFC (Kritzer and Creutz, 2008), higher baseline spine density in the nucleus accumbens (McGrath and Briand, 2022), and ∼10% less D1 receptors in the striatum (Levesque et al., 1989), as compared to males. Furthermore, multiple studies have revealed sex-specific stress-related developmental effects (Caruso et al., 2018; McGrath and Briand, 2022; Tan et al., 2021). For instance, social isolation has been found to induce a greater increase in spine density in the infralimbic cortex of females following postweaning social isolation (McGrath and Briand, 2022), and social isolation during adolescence has been associated with decreased social behavior in females and increased aggression in males, which appear to be linked to opposing sex-specific activity patterns with either increased or decreased PFC activity in males or females, respectively (Tan et al., 2021). It has also been noted that genetic differences may predispose certain rat strains to exhibit more predominant sex differences (Rivera-Garcia et al., 2020). Together, these findings suggest that social isolation likely impacts females or subjects of different genetic backgrounds differentially based on baseline dopamine signaling components. Thus, it will be important in future studies to better understand which processes are conserved or differ based on such underlying factors.

## 5 Conclusions

Taken together, the findings reported herein provide a mechanistic understanding of the critical effects of social isolation postweaning and during adolescence on reward-related dopamine dynamics in the mPFC and NAc. It will be of interest in future studies to determine if other external stimuli or mitigating factors may reverse these deficits at a specific developmental stage following such social isolation. Finally, it will be important to examine the impact of the isolation-induced changes in dopamine signaling for its effects on other outcome measures. For instance, the COVID pandemic has been linked to an increased incidence of eating disorders, such as anorexia nervosa, in adolescent children (Agostino et al., 2021; Devoe et al., 2023; Haripersad et al., 2021), and it has been established that loneliness and eating disorders are bidirectionally related (Cortes-Garcia et al., 2022). Given our observed link between reward-related feeding behaviors and dopamine changes in the mPFC and NAc during social isolation, it is possible that there is a mechanistic link between the increased incidence of eating disorders stemming from the pandemic lockdowns and lack of social interaction (e.g., home confinement vs. traditional school settings) with dopaminergic dysregulation, a contention that will need to be more fully addressed in future studies.

## Funding and disclosure

The authors have no competing financial interests in relation to the work described. This work was supported by grants from the NIH National Institute on Drug Abuse (R01 DA051831 and U01 DA053826 to CDF) and 10.13039/100005188Tobacco-Related Disease Research Program (TRDRP T30FT0967 to VL). CC and GC were supported by the European Erasmus Globus Fellowship through the University of Cagliari.

## CRediT authorship contribution statement

**Valeria Lallai:** Conceptualization, Data curation, Formal analysis, Funding acquisition, Investigation, Methodology, Project administration, Supervision, Validation, Visualization, Writing – original draft, Writing – review & editing. **Cristina Congiu:** Data curation, Formal analysis, Investigation, Methodology, Validation, Visualization, Writing – original draft, Writing – review & editing. **Giulia Craig:** Data curation, Formal analysis, Investigation, Methodology, Validation, Visualization, Writing – original draft, Writing – review & editing. **Letizia Manca:** Data curation, Investigation, Methodology, Writing – original draft, Writing – review & editing. **Yen-Chu Chen:** Investigation, Writing – review & editing. **Angeline J. Dukes:** Investigation, Writing – review & editing. **Christie D. Fowler:** Conceptualization, Data curation, Formal analysis, Funding acquisition, Methodology, Project administration, Resources, Supervision, Validation, Visualization, Writing – original draft, Writing – review & editing. **Laura Dazzi:** Conceptualization, Data curation, Formal analysis, Funding acquisition, Methodology, Project administration, Resources, Supervision, Validation, Visualization, Writing – original draft, Writing – review & editing.

## Declaration of competing interest

None

## Data Availability

Data will be made available on request.
